# *CACNA1C rs1006737*, Threatening Life Events, and Gene–Environment Interaction Predict Major Depressive Disorder

**DOI:** 10.3389/fpsyt.2019.00982

**Published:** 2020-01-22

**Authors:** Mingzhe Zhao, Jiarun Yang, Xiaohui Qiu, Xiuxian Yang, Zhengxue Qiao, Xuejia Song, Lin Wang, Erying Zhao, Yanjie Yang, Depin Cao

**Affiliations:** ^1^ Psychology Department of the Public Health Institute of Harbin Medical University, Harbin, China; ^2^ Department of Health Management of Harbin Medical University, Harbin, China

**Keywords:** *CACNA1C*, polymorphism, threatening life events, gene–environment interaction, major depressive disorder

## Abstract

**Introduction:**

*CACNA1C rs1006737* is a novel variant in discovery of replicable associations in major depressive disorder (MDD). However, there have been no specific studies considered effect of environmental pathogens to date examining its clinical significance. In this study we investigated the interaction effect between *CACNA1C rs1006737* polymorphism and threatening life events (TLEs) in MDD and carried out a meta-analysis of published findings.

**Methods:**

A total of 1,177 consecutive participants were genotyped. Information on exposure to TLEs, socio-demographic data, and history of psychological problems among first-degree relatives was collected. MDD was diagnosed according to the Chinese version of the 24-item Hamilton Rating Scale for Depression.

**Results:**

There was a significant interaction effect between *CACNA1C rs1006737* polymorphism and TLEs in MDD. A dose–response relationship was found between *CACNA1C rs1006737* genotypes and TLEs in MDD. The results of the meta-analysis showed that *CACNA1C rs1006737* genotypes interacted with TLEs in MDD.

**Conclusion:**

*CACNA1C rs1006737* genotype and previous exposure to TLEs interact to influence the risk of developing MDD. We propose that *CACNA1C rs1006737* may represent a target for novel pharmacological therapies to prevent or treat MDD.

## Introduction

Predisposition to complex diseases is not solely conferred by genetic factors; it is also influenced by environmental exposure. Delineating their respective contributions is a major challenge in the study of complex diseases (1–5). Gene–environment interactions (G × E) are thought to account for a large fraction of the unexplained variance in heritability and disease risk (6, 7). However, disease risk due either to environmental exposure and/or its interactions with genotype remains poorly understood (8, 9).

Major depressive disorder (MDD) is an example of a complex disease for which G × E are likely important. An early study on G × E in MDD showed that a length polymorphism (*SLC6A4*) in the promoter region of the *5-HTT* gene mediates the response to stressful life events (10). The *rs1006737* polymorphism in the third intron of the gene encoding the Cav1.2 subunit of the L-type voltage-gated calcium channel gene (*CACNA1C*) (Chromosome12:2345295), which is highly expressed throughout the forebrain (11), has been attributed to G × E. Cav1.2 couples a transient increase in membrane permeability to cell membrane depolarization and gene transcription and plays a critical role in dendritic development, neuronal survival, synaptic plasticity, memory formation, learning, and behavior (12–16). *In vitro* studies have shown that disease-associated increases in Ca^2+^ influx *via* Cav1.2 channels can alter gene expression (17) and contribute to activity-dependent dendrite retraction (18), which can occur in response to chronic stress (19).

Threatening life events (TLEs) precede the onset of depressive episodes more frequently than expected by chance (20); TLEs were shown to cluster before the onset of a depressive episode or an exacerbation of symptoms (21, 22). Although a single stressor may have relatively minor effects, the cumulative effects of multiple stressors (23) can lead to psychiatric disorders, consistent with a dose–response effect (24).

It was recently reported that the *CACNA1C rs1006737* polymorphism mediates the influence of TLEs on human MDD (25). However, this has been contradicted by another study (26). Neither of these investigations addressed the specificity of the *CACNA1C rs1006737* and dose-response effects of TLEs. Examining the perceived threat level of TLEs in the context of *CACNA1C rs1006737* genotype may provide more detailed insight into the nature of genetic effects on stress response.

Measurements of environmental risk can vary across studies; a meta-analysis is one tool for determining whether a result transcends inter-study variation, and is widely used in the field of psychiatric genetics, which has been plagued in recent years by non-reproducibility (27). By pooling data from several studies, a meta-analysis maximizes the power to detect significant effects and avoids overemphasizing estimates from any single study (28).

In this report, we investigated G × E effects between *CACNA1C rs1006737* genotype, TLEs, and MDD in a large clinical sample. A meta-analysis was also carried out to evaluate the current evidence for these interactions.

## Material and Methods

### Study Population

From November 2014 and December 2017, 590 patients with MDD (420 women and 170 men) were recruited for the study (mean age: 44.22 ± 13.45 years) along with 587 age- and sex-matched control subjects without a history of neuropsychiatric disorders. Both patients and controls subjects were from the same geographic area in Northern China and were of Chinese Han ethnicity, and provided written, informed consent before participation in the study. The study was approved by the Ethics Committee of Harbin Medical University.

### Independent Measures

Participants completed three questionnaires: a socio-demographic questionnaire, the Chinese version of the 24-item Hamilton Rating Scale for Depression (HRSD-24), and the Life Events Scale (LES). The socio-demographic questionnaire was used to collect detailed information about socioeconomic background and medical history including individual and family psychiatric history. The HRSD-24 is a reliable tool that has been used in several studies to assess depressive symptoms (29–31). Patients above the threshold (21 points) were included in the study. The LES was used to evaluate negative life events; this self-rating questionnaire consists of 48 items in three areas—i.e., family life (28 items), work-related problems (13 items), and social and other aspects (seven items) (32).

### Genotyping

Genomic DNA was extracted from venous blood samples using the AxyPrep Blood Genomic DNA Miniprep kit (Axygen, Union City, CA, USA) and the single nucleotide polymorphism (SNP) rs1006737 of the *CACNA1C* gene was detected by PCR amplification using primers designed with Primer 5.0 software, which had the following sequences: 5'-AAGTTCCATTCCATCTCAGCCCGAA-3' (forward) and 5'-TGTTTTCAGAGCCGGAGACCTCACA-3' (reverse). SNP analysis was performed using SNaPshot according to the manufacturer's instructions.

### Statistical Analysis

Data were analyzed using R Studio 1.1.423. The χ^2^ test was used to evaluate differences in the distributions of independent variables. Genotype frequencies were tested for Hardy–Weinberg equilibrium. The Bonferroni method was used for multiple-testing correction of genetic association in univariate analysis and the significance level was set at P <0.01 (0.05/5). G × E were examined with a logistic regression model. Four predictor variables were used: *CACNA1C* rs1006737 genotype (*GG*, *GA*, or *AA*), sex, family history, and either the presence/absence or number of TLEs. The dependent variable was the onset of an episode of MDD. Study power was calculated with QUANTO 1.2.4 (http://hydra.usc.edu/gxe/).

For analyses incorporating TLEs, the TLEs were coded so that 0 represented no TLE occurrence and values of 1, 2, 3, or ≥4 represented the occurrence of TLEs that were minor, low-moderate, high-moderate, and severe, respectively. To simplify the interpretation of interactions, the number of TLEs was coded using four dummy variables (X1, X2, X3, and X4). If there was no TLE, all four were coded as zero. For example, if there was one, two, or three TLEs, X1, X2, and X3, respectively, were coded as 1. Thus, the coding for three TLEs was: X1 = 1, X2 = 1, X3 = 1, and X4 = 0. This method of coding dummy variables known as thermometer coding does not alter the model results but is simpler yet mathematically equivalent to contrasts (33); compared to typical indicator variables, it greatly simplifies the model selection process. Removing a level of a standard indicator variable requires recoding the data and a likelihood ratio test; with thermometer coding, the task is not different from removing other independent variables.

We conducted a meta-analysis by pooling results from previous G × E studies of the *CACNA1C rs1006737* polymorphism, TLEs, and MDD with findings from the present study. The Lipták–Stouffer z-score approach was used to obtain an aggregate value for the significance level of tests weighted by sample size, and a sensitivity analysis was conducted by recomputing effect size after systematically removing each study in turn. To gauge potential publication bias, we calculated fail-safe N and its ratio (34, 35).

## Results

### Frequencies of Independent Variables

Demographic and genotypic data for the study population are shown in **Table 1**. About half of subjects had TLEs, and one in nine had a family history of psychological problems among first-degree relatives. Approximately half of subjects had the *G/G* genotype, one in three the *G/A* genotype, and the remaining subjects the *A/A* genotype. Genotype frequencies were in Hardy–Weinberg equilibrium among both cases and controls.

**Table 1:** Summarized frequencies of socio-demographic and independent variables.

Variables	Frequencies
Socio-demographic variables	
Gender	
Female	802 (68.1%)
Male	375 (31.9%)
Mean age	43.6 (s.d. 11.55)
Independent variables	
* CACNA1C*(rs1006737) genotypes	
* G/G*	770 (65.4%)
* G/A*	365 (31.1%)
* A/A*	42 (3.5%)
Exposure to threatening experiences	
No TLE	592 (50.2%)
1 TLE	284 (24.2%)
2 or more TLEs	301 (25.6%)
Family history of psychological problems among first-degree relatives	
FH+	102 (9%)
FH–	1,075 (91%)

FH, family history; s.d., standard deviation; TLE, threatening life events.

### Associations With MDD

There were significant differences in genotype (χ^2^ = 6.36, P = 0.04), homozygosity (χ^2^ = 5.47, P = 0.01), TLEs (χ^2^ = 64.27, P = 1.105e^−14^), and family history (χ^2^ = 66.55, P = 3.408e^−16^) distribution between patients with MDD and controls (**Table 2**). TLEs (P < 0.01) and family history (P < 0.01) were still associated with MDD after Bonferroni correction. On the basis of sample size of the study, the power for association study of *CACNA1C rs1006737* was 98.92%.

**Table 2:** Association between depression and genetic or environmental factors.

	Case	Control	χ^2^	df	*P*
Genotypes			6.36	2	0.04
* G/G*	384 (65)	386 (66)			
* G/A*	177 (30)	188 (32)			
* A/A*	29 (5)	13 (2)			
Homozygous			5.47	1	0.01
G/*	561 (95)	574 (98)			
* A/A*	29 (5)	13 (2)			
Alleles			1.08	1	0.29
* G*	945 (80)	960 (82)			
* A*	235 (20)	214 (18)			
Threatening life events			64.27	2	1.105e−14*
No	229 (39)	363 (62)			
1	167 (28)	117 (20)			
2 or more	194 (33)	107 (18)			
Family history			66.55	1	3.408e−16*
Negative	499 (85)	576 (98)			
Positive	91 (15)	11 (2)			

*P <0.01 after Bonferroni correction.

### Interaction Between TLE Occurrence and *CACNA1C rs1006737* Genotype in the Prediction of MDD

In our initial analyses, which considered only the presence or absence of TLEs, a full model was first generated including *GG*/*GA*/*AA* genotype, sex, family history, and the occurrence of TLEs. We then selected the optimal model based on the Akaike information criterion. The dominant mode of action that combined the effects of *GA* and *AA* genotypes showed an improvement in fit. This best-fit model suggested significant main effects of family history (β = 8.56, s.e. = 0.37, P = 1.29e^−08^) and TLE occurrence (β = 2.08, s.e. = 0.15, P = 1.26e^−06^) but not of sex (β = 0.83, s.e. = 0.13, P = 0.17) or genotype (β = 0.91, s.e. = 0.17, P = 0.55) for predicting MDD (**Table 3**). However, a significant genotype × TLE interaction was found (β = 1.81, s.e. = 0.27, P = 0.02). Estimates based on this model indicated that family history and TLE exposure could influence the prediction of MDD, and that genetics alone cannot predict MDD but can modify the risk effect conferred by exposure to TLEs. On the basis of sample size of the study, the power was 87.59% to detect a significant effect of rs1006737× TLE interaction on MDD under dominant genetic model.

**Table 3:** *CACNA1C* genotype interaction with threatening life experiences.

	β （95% CI）	SE	P
Sex	0.83 (0.63–0.99)	0.13	0.17
Family history	8.56 (4.34–19.46)	0.37	1.29e−08
No/Any TLE (E)	2.08 (1.55–2.81)	0.15	1.26e−06
*CACNA1C* genotype (*G*)	0.91 (0.64–1.26)	0.17	0.55
Gene (*G*) × Environment (E)	1.81 (1.06–3012)	0.27	0.02

### Interaction Between Number of TLEs and *CACNA1C rs1006737* Genotype in the Prediction of MDD

Based on the evidence for an interaction between *CACNA1C rs1006737* genotype and TLE exposure in the prediction of MDD, we explored how this polymorphism alters the dose-response relationship between number of TLEs and risk for MDD onset.

We generated a full model encompassing the dominant action model (*GG* vs. *GA/AA*), TLEs, sex, and family history. Two of the four possible interactions with genotype and number of TLEs were retained. The final model for the prediction of MDD included sex, family history, minor threat, low-moderate threat, high-moderate threat, main effects of *CACNA1C rs1006737* genotypes, and the interaction between genotype and TLE values of 1 and 3 (**Table 4**).

**Table 4:** *CACNA1C* genotype interactions with threat exposure.

	β （95% CI）	SE	P
Sex	0.84 (0.65–1.09)	0.13	0.20
Family history	8.35 (4.22–19.01)	0.37	2.05e−08
Minor threat	1.81 (1.26–2.59)	0.18	0.001
Low-moderate threat	0.85 (0.51–1.45)	0.26	0.56
High-moderate threat	2.16 (1.18–3.97)	0.31	0.01
*CACNA1C* genotype (*G*)	0.90 (0.64–1.26)	0.17	0.55
Gene (G) × Minor threat	2.02 (1.03–4.06)	0.34	0.04
Gene (G) × Low-moderate threat	1.54 (0.56–4.39)	0.52	0.40
Gene (G) × High-moderate threat	0.28 (0.08–0.93)	0.61	0.03

The main effects of *CACNA1C rs1006737* genotypes (β = 0.90, s.e. = 0.17, P = 0.55), sex (β = 0.84, s.e. = 0.13, P = 0.20), and a TLE of 2 (β = 0.85, s.e. = 0.26, P = 0.56) were non-significant in this final model. Conversely, those of a TLE of 1 (β = 0.1.81, s.e. = 0.18, P = 0.001) or 3 (β = 2.16, s.e. = 0.31, P = 0.01) and family history (β = 8.35, s.e. = 0.37, P = 2.05e^−08^) were significant. Importantly, we found that the *CACNA1C rs1006737* genotype interaction with a TLE of 1 (β = 2.02, s.e. = 0.17, P = 0.04, γ = 2.37) was significant, with individuals harboring the *AA* and *GA* genotypes showing greater sensitivity to the depression-inducing effects of a TLE of 1 than those with the GG genotype. We also observed a significant interaction between genotype and a TLE of 3 (β = 0.28, s.e. = 0.61, P = 0.03, γ = 0.13)—that is, high exposure to TLEs was associated with an increase in risk for MDD in all genotypes. Interaction coefficients (γ) ranging from a TLE of 1 to 3 indicated a low exposure–gene effect between exposure level and *CACNA1C rs1006737* polymorphism in MDD. In contrast, the interaction between *CACNA1C rs1006737*genotypes and a TLE of 2 (β = 1.54, s.e. = 0.52, P = 0.40) was non-significant in this final model (**Table 4**). Estimates based on this model indicated that family history and TLE exposure still have main effects on the prediction of MDD, and that genetics can modify the risk effect conferred by exposure to TLEs from a minor threat to a high-moderate threat.

### Meta-Analysis

**Figure 1:**
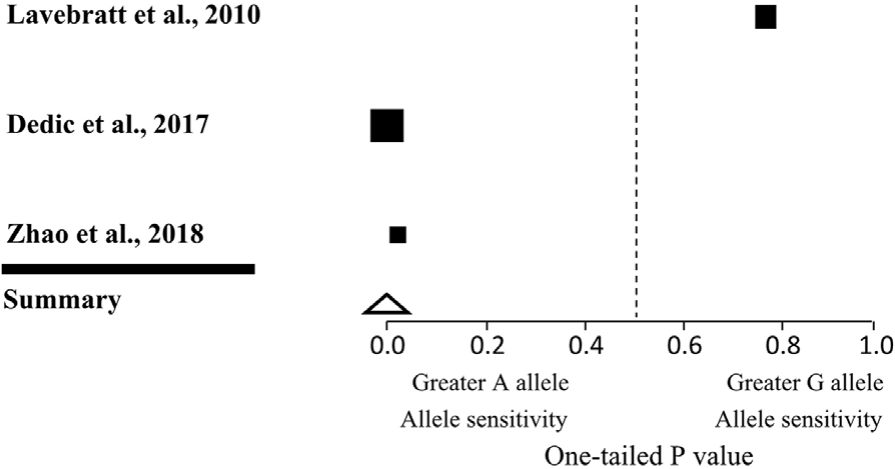
Forest plot of three human observational studies on the interaction effect of *CACNA1C* genotype and life stress on depression. Squares indicate the one-tailed P value for each study, with lower values denoting greater stress sensitivity of *A* allele carriers and higher values corresponding to greater stress sensitivity of *G* allele carriers. The size of the box reflects the relative sample size. The triangle indicates the overall result of the meta-analysis.

We included studies in our meta-analysis that met three criteria: the study had to be published in a peer-reviewed journal, and include genotypic information on the *CACNA1C rs1006737* gene as well as a measure of TLEs. After searching the PubMed, Wolters Kluwer, and Web of Science databases, we identified two previous studies that met all three criteria (8.9). The results of these studies were pooled with the present findings to assess the interaction between *CACNA1C rs1006737* polymorphism and TLEs in MDD in a total of 8,728 subjects (**Table 5** and **Figure 1**). The significance of the results remained robust in the sensitivity analysis when each study was removed in turn, with the exception of one study (0.0001 < P < 0.01). To render the outcome in the analysis non-significant (P = 0.05), an additional four unpublished or undiscovered studies with average sample size of n = 2909 and a non-significant result (P = 0.50) would be required. This yielded a fail-safe ratio of one study excluded for each one included in the meta-analysis.

**Table 5:** Studies on the interaction between *CACNA1C* polymorphism, threatening life events, and depression included in the meta-analysis.

Source, year	No. of Subjects	1-Tailed P Value	P Value After Study Exclusion
Lavebratt et al. (26)	2,743	0.77	0.0001
Dedic et al. (25)	4,808	0.001	0.41
Zhao et al. (34)	1,177	0.01	0.01
**Total:**	**8,728**		
**Average sample size:**	**2909**	**0.003**	

P = 0.003 stands for overall effect size which is significant.

## Discussion

Prior studies documenting the interaction effect between *CACNA1C rs1006737* genotypes and TLEs in MDD have reported a positive interaction effect (25) or contrary findings (26). However, neither of these studies focused on a single marker, resulting in low *a priori* probability and power. Additionally, the influence of the *CACNA1C rs1006737* polymorphism on the dose-response relationship between TLEs and risk for MDD was not reported in either study. Here we attempted to replicate the prior finding that *CACNA1C rs1006737* genotypes modified the depressogenic effects of TLEs. Our second goal was to clarify the dose-response relationship between TLEs and *CACNA1C rs1006737* genotypes in MDD. Finally, we carried out a meta-analysis of the interaction between *CACNA1C rs1006737*genotypes and TLEs in MDD to evaluate the current evidence.

Our main findings were that *CACNA1C rs1006737* genotypes and TLEs were independently associated with MDD, and that *CACNA1C rs1006737* genotypes significantly modified the risk conferred by TLEs for MDD; moreover, a dose-response relationship was found to exist between *CACNA1C rs1006737* genotypes and TLEs in MDD, with the meta-analysis confirming an interaction between these variables.

Previous association studies of MDD have suffered from low rates of reproducibility. Instead of a main effect of genotype on phenotype, G×E effects were reported. Since the latter are more difficult to detect than the former (36, 37), replications may be expected to be more rare and have a specific value when they do occur. We first analyzed the interaction between TLE occurrence and *CACNA1C rs1006737* genotypes in the prediction of MDD and found a better-fitting model after adjusting for potential confounds such as sex and family history. There is conflicting evidence regarding the impact of sex on depressive disorder, with some studies reporting it as valid for both sexes (38–41), and others suggesting an effect only in women (42, 43) or the inverse effect in men (44). We found a significant sex difference between patients with MDD and controls. Family history of psychological problems is associated with both exposure (45) and outcome (46); we also found a significant difference in family history between patients with MDD and controls, and therefore included this parameter in the model. In the regression analysis, family history remained significant but not for sex and TLEs, and had a main effect but not for genotype. Importantly, a genotype × TLE interaction was observed. These findings suggest that the *CACNA1C rs1006737* polymorphism does not have a main effect on MDD by itself, but does in combination with TLEs.

We then analyzed the interaction between number of TLEs and *CACNA1C rs1006737* genotypes in the prediction of MDD. Sex and family history were retained in the model, although the effect of sex was non-significant. Our results showed that individuals with the *AA* or *GA* genotype had greater sensitivity to the depressogenic effects of a TLE of 1 than those with the *GG* genotype, and that high exposure to TLEs was associated with a marked increase in the risk for MDD for all genotypes. The dose–response effect analysis revealed a low exposure-gene effect. Exposure levels were measured based on retrospective reporting by participants, and bias in this data could have influenced the dose-response effect. In many cases, cumulative measurements can be obtained by making repeated measurements over time, which enhances power to detect G × E (47). The dose–response effect in our study suggests that elucidating the mechanism underlying the progression from genetic variation to MDD requires more precise measures of environmental risk factors and stressful experiences.

A strength of this study is that it included a meta-analysis as well as an analysis of original data. The former provided evidence of a *CACNA1C rs1006737* genotype × TLE interaction effect in MDD. However, our previous meta-analysis of G × E (34, 35) showed that a subgroup analysis stratified by type of stressor, study design, or subjects' ancestry should be carried out wherever possible in order to reduce confounds for the G × E effect.

Our study also had some limitations. Firstly, data on environmental pathogens were collected from subjects' retrospective reports, which has risks such as forgetting, revisionist recall, and bias due to cognitive dysfunction or low mood (48). Secondly, mistreatment in childhood was not considered as an independent environmental risk factor separate from TLEs although it can affect the development of neural circuitry and is therefore a good candidate to study G × E effects in mental disorders (49, 50).

In conclusion, we provide evidence supporting an effect modification by the *CACNA1C rs1006737* genotype on the risk of MDD conferred by previous exposure to TLEs. Thus, *CACNA1C rs1006737* is an example of a gene that influences vulnerability to MDD not by a main effect on risk but rather by modulating sensitivity to the negative effects of the environment. Future work will include genome-wide association studies data to test for G × E interactions.

## Data Availability Statement

The datasets for this article are not publicly available because the datasets were also used in another study which is not published yet. Request to access the datasets should be directed to YY, yanjie1965@163.com.

## Ethics Statement

The studies involving human participants were reviewed and approved by Ethics Committee of Harbin Medical University. The patients/participants provided their written informed consent to participate in this study. Written informed consent was obtained from the individual(s) for the publication of any potentially identifiable images or data included in this article.

## Author Contributions

MZ and JY conducted the statistical analyses and wrote the first draft of the manuscript. XQ and XY provided expertise in MDD search. ZQ and XS collected the data. LW and EZ did the experiment. YY and DC designed this study and provided expertise. All authors were involved in modifying the secondary-analysis design and editing the manuscript. All authors contributed to and have approved the final manuscript.

## Funding

This study was supported by the National Natural Science Foundation of China (81473054, 81773536) to YY.

## Conflict of Interest

The authors declare that the research was conducted in the absence of any commercial or financial relationships that could be construed as a potential conflict of interest.
